# F162. MORE EFFORT, BUT LESS EFFICIENCY - WORKING MEMORY IN SCHIZOPHRENIA: AN INVESTIGATION INTO CAUSE FOR DIVERGENT RESULTS

**DOI:** 10.1093/schbul/sby017.693

**Published:** 2018-04-01

**Authors:** Pavan Mallikarjun, Sabrina Meecham, Rachel Upthegrove, Paris Alexandros Lalousis, Anna Diukova, Dorothee Auer, Peter Liddle

**Affiliations:** 1 University of Birmingham; 2 Birbeck, University of London; 3 Cardiff University; 4 University of Nottingham

## Abstract

**Background:**

Functional brain imaging studies of working memory in people with schizophrenia have reported divergent reports with some studies reporting hypofrontal activation while report hypofrontal activation. The inconsistencies in the literature have been postulated to be related to task requirements or poor task performance in schizophrenia. An alternative hypothesis proposed is that different regions within the PFC show either hypo or hyperactivity, depending on their location. In view of the inconsistencies in previous research, the aim of the current research is to examine abnormalities in a cohort of partially remitted people with schizophrenia and matched controls

**Methods:**

Twenty people with schizophrenia and 20 matched healthy controls (matched on age, gender and parental social and economic status) were selected from the Nottingham area to undergo a functional magnetic resonance imaging (fMRI) scan while undergoing two levels of the visual n-back task (zeroback and two back). The behavioural results (latency and accuracy) on the nback task was compared using repeated measures analysis. The MR images were reoriented, realigned, co-registered to the T1-weighted anatomical image, and normalised to the MNI space. Normalised images were resliced to a voxel size of 3 mm3, smoothed with an 8 mm full-width half-maximum Gaussian kernel using SPM 5. The GLM approach implemented in SPM 5 was utilized for fMRI data modeling and analyses. Each epoch type (zero-back, two-back and rest) was modeled in a boxcar design, using a canonical hemodynamic response function and its temporal derivative. The single group second level analysis consisted of voxelwise one-sample t-tests with significance threshold set at voxel level p < .05, false discovery rate (FDR) corrected for multiple comparisons across the entire brain.

The between-group comparison employed voxelwise two sample t-tests with the significance criterion based on the spatial extent of suprathreshold voxel clusters, a method proposed by (Friston et al., 1994). The criterion for inclusion of a voxel in a cluster was set at p < .05 (uncorrected) and a cluster was considered as significant at level of p < .05 (corrected).

**Results:**

Patients exhibited relatively high general function and mild symptoms, but had significantly longer reaction time and decline in performance. Both controls and patients activated similar set of brain regions similar to Central executive network and deactivated brain areas comprising the default mode network. Most noteworthy, we did not find evidence for hypofrontality (reduced activity in DLPFC in patients compared to controls) in this study. Compared to controls, patients showed areas of greater activation in left postcentral gyrus, anterior and posterior medial frontal gyrus and left superior temporal gyrus.

**Discussion:**

Both controls and patients showed activation in widespread brain areas consistent with the CEN, and deactivation in areas consistent with DMN regions. There was no evidence of hypofrontality in this sample of remitted people with schizophrenia. Patients showed activation patterns on the zeroback task similar to the activation patterns for controls on the two back task, suggesting that patients showed increased brain activation on a task with lesser requirements, though behavioural results suggest inefficiency despite the increased effort.

